# Collateral vessels on magnetic resonance angiography in endovascular-treated acute ischemic stroke patients associated with clinical outcomes

**DOI:** 10.18632/oncotarget.21081

**Published:** 2017-09-19

**Authors:** Liang Jiang, Hao-Bo Su, Ying-Dong Zhang, Jun-Shan Zhou, Wen Geng, Huiyou Chen, Quan Xu, Xindao Yin, Yu-Chen Chen

**Affiliations:** ^1^ Department of Radiology, Nanjing First Hospital, Nanjing Medical University, Nanjing, China; ^2^ Department of Vascular and Interventional Radiology, Nanjing First Hospital, Nanjing Medical University, Nanjing, China; ^3^ Department of Neurology, Nanjing First Hospital, Nanjing Medical University, Nanjing, China

**Keywords:** acute ischemic stroke, collateral vessels, endovascular recanalization, MRI

## Abstract

**Purpose:**

Collateral vessels were considered to be related with outcome in endovascular-treated acute ischemic stroke patients. This study aimed to evaluate whether the collateral vessels on magnetic resonance angiography (MRA) could predict the clinical outcome.

**Materials and Methods:**

Acute stroke patients with internal carotid artery or middle cerebral artery occlusion within 6 hours of symptom onset were included. All patients underwent MRI and received endovascular treatment. The collateral circulations at the Sylvian fissure and the leptomeningeal convexity were evaluated. The preoperative and postoperative infarct volume was measured. The clinical outcome was evaluated by mRS score at 3 months after stroke.

**Results:**

Of 55 patients, Cases with insufficient collateral circulation at the Sylvian fissure and leptomeningeal convexity showed that the NIHSS score at arrival and preoperative infarct volume were significantly lower in mRS score of 0–2 (both *P* < 0.05) than mRS score of 3–6. Multivariate testing revealed age and collateral status at the leptomeningeal convexity were independent of the clinical outcome at 3 months after stroke (odds ratio (95% confidence interval): 1.094 (1.025–1.168); 9.542 (1.812–50.245) respectively). The change of infarct volume in the group with mRS score of 0–2 was smaller than that with mRS score of 3–6. While multivariate logistic models showed that postoperative infarct volume was non-significant in predicting the clinical outcome after stroke.

**Conclusions:**

The extent of collateral circulation at the leptomeningeal convexity may be useful for predicting the functional recovery while the relationship between postoperative infarct volume and clinical outcome still requires for further study.

## INTRODUCTION

Acute ischemic stroke (AIS) is the most common type of stroke, accounting for about 80% of the total cerebral apoplexy [1]. It is the third leading cause of death worldwide. In recent years, the incidence of AIS is increasing and often leads to devastating motor disability [2–4], especially the AIS caused by the middle cerebral artery occlusion. As a new method for treatment of AIS, endovascular recanalization can increase the recanalization of occluded cerebral arteries quickly and significantly [5–9]. The latest clinical practice guideline recommends an endovascular recanalization strategy for AIS if the intervention can be applied within 6 hours from last seen normal [10]. AIS outcome varies considerably due to the compensatory ability of the collateral circulation and the ensuing cerebral blood flow [11, 12]. Good angiographic collaterals have been associated with improved recanalization [13].

Multi-parametric magnetic resonance imaging (MRI), including diffusion-weighted imaging (DWI), perfusion-weighted imaging (PWI), and magnetic resonance angiography (MRA), has increasingly been used to optimize the therapy strategy after stroke [14, 15]. As a noninvasive imaging method, MRI has also become a recent trend to visualize collaterals [16]. The primary collateral circulation is occasionally established via leptomeningeal anastomoses from the anterior cerebral artery and posterior cerebral artery (PCA) when the proximal middle cerebral arteries (MCA) occlusion. Prominent PCA laterality on three-dimensional time-of-flight (3D-TOF) MRA was reported as a MRI radiologic marker of collateral flow [17, 18]. Good collateral circulation could independently predict good outcome in AIS [19, 20]. Poor arterial collateralization was associated with poor outcome after recanalization success [21]. A favorable arterial collateralization as determined by leptomeningeal anastomoses may improve post-revascularization outcome [12, 13].

Previous experimental studies showed that symptom onset to recanalization, initial stroke severity at presentation in the form of the National Institutes of Health Stroke Scale (NIHSS) or infarct volume on imaging play a role in influencing ultimate clinical outcome [22, 23]. In this study, we focused on the association between the baseline collateral status on MRA and stroke outcomes after endovascular recanalization. In addition to investigate the effects of the infarct volume on the initial collateral flow and the final functional status, we also compared the collateral circulation, infarct volume between pre and post treatment.

## MATERIALS AND METHODS

### Patient selection and evaluation

This was an observational and prospective study. Our analysis was performed on data collected from a prospective registry of patients who were eligible for recanalization therapy for acute infarct of MCA and/or internal carotid artery (ICA). The stroke patients who admitted to Nanjing First Hospital between October 2015 and March 2017 were analyzed. The inclusion criteria for this study were: (1) ischemic stroke with relevant infarction that was documented by relevant neuroimaging studies, (2) presentation within 6 hours of symptom onset and receive endovascular recanalization treatment, (3) severe stenosis or occlusion in the ICA or proximal MCA including the main stem (M1) or secondary trunks (M2). The following exclusion criteria were also applied: (1) missing modified Rankin Scale (mRS) score at 3 months after stroke, (2) no non-contrast CT and MRI examination before the endovascular recanalization therapy, or the MRI which has motion artifact was unable to evaluate. (3) hemodynamically irrelevant stenosis or a bilateral steno-occlusion that prevented measurement of the collateral status, (4) no endovascular recanalization treatment. Finally a total of 55 cases are eligible for the current analysis according to the inclusion and exclusion criteria. Acute stroke management was performed according to the current clinical practice guidelines for stroke care, institutional protocols, and at the discretion of individual physicians with direct liability [24, 25]. The local Institutional Review Board approved this study and all patients gave their consent to participate in the study.

We evaluated all study participants according to a protocol that included baseline demographic and clinical information, including age, sex, history of previous stroke, hypertension, diabetes mellitus, hyperlipidemia, atrial fibrillation, habitual smoking, the NIHSS on admission, and mRS score at 3 months after the onset of symptoms. Good clinical outcome was defined as a mRS score ≤ 2 at 3 months [26].

### CT, MRI methods and image analysis

Non-enhanced CT image was acquired according to standard departmental protocols with a 16-channel multidetector CT scanner (Sensation 16, Siemens, Germany). Non-enhanced CT was performed with the patient in head holder in the transverse plane with minimal variations between scanners, and with scanning parameters of 120 kVP, 250 mAS, and 5-mm section thickness. MRI was performed using a 3.0 T unit (Ingenia; Philips Medical Systems, Netherlands). The MRI protocol included the fluid-attenuated inversion recovery (FLAIR), DWI and 3D-TOF MRA. FLAIR MRI parameters were obtained axially using inverse recovery (IR) sequence as follows: repetition time (TR), 7000 ms; echo time (TE), 120 ms; acquisition matrix, 356*151; field of view (FOV), 230 mm*230 mm ;flip angle (FA), 90^°C^; slices, 18; section thickness, 6 mm; and intersection gap, 1.3mm. DWI was performed using spin echo (SE) sequence, the following parameters: TR, 2501 ms; TE, 98 ms; acquisition matrix, 152*122; 3 directions; FOV, 230 mm*230 mm; FA, 90°C; slices, 18; section thickness, 6mm; and intersection gap, 1.3 mm. DWI was obtained with b values of 0 and 1000 s/mm^2^. 3D-TOF MRA were obtained using fast field echo (FFE) sequence, parameters were as follows: TR, 22 ms; TE, 3.5 ms; acquisition matrix, 400*232; FOV, 240 mm*240 mm; FA, 18^°C^; slices, 140; section thickness, 20 mm; and intersection gap, 10 mm.

Collateral status was measured primarily using a method proposed by Maas et al. [27]. The collateral vessels in the Sylvian fissure and leptomeningeal convexity were assessed in the contrast-enhanced MRA images of 2-mm section separately. The collateral vessels of the occluded hemisphere mainly compared with the patent hemisphere, as follows: 1, absent; 2, less than the patent contralateral side; 3 equal to the patent contralateral side; 4 greater than the patent contralateral side; and 5, exuberant. The collateral status was separately categorized into insufficient (grade ≤ 2) and sufficient (grade ≥ 3). Hyperintense vessels (HV) were defined as a linear or curved hyperintensity on FLAIR MRI corresponding to a typical arterial course. Ten imaging slices, from the first M1 MCA appearance rostrocaudally to the tenth image, were analyzed and one or more HVs recognized on one slice were rated one point. The resulting HV score ranged from 0 to 10. Two neuroradiologists (H-B S and XY) assessed the collateral status and HV. In case of a discrepant score between the two readers, images were reviewed and a consensus was established.

DWI lesion volume measurements were performed by the author who was blinded to the clinical information. DWI lesion volume was measured semiautomatically using Philips workstation (IntelliSpace Portal). Two reviewers who were blinded to the study purpose and clinical characteristics assessed the reperfusion status using the modified Thrombolysis in Cerebral Infarction (TICI) score in postrecanalization conventional angiography images [28].

### Statistical analyses

Continuous data are shown as the mean ± SD, whereas categorical variables are presented as absolute and relative frequencies. We analyzed differences between groups using chi-squared test for categorical variables and the independent-samples *t*-test or Fisher's exact test for continuous variables. In addition, multivariate logistic regression analysis was performed to predict the independent contribution of factors in clinical outcome. *P* < 0.05 was considered statistically significant. All statistical analyses were conducted using commercially available software (SPSS for Windows, version 19.0; SPSS).

## RESULTS

A total of 55 patients were included in this study: 33 men and 22 women with a mean age of 68.96 ± 13.54 years (range, 32–89 years). The median NIHSS score at arrival was 12.71 ± 5.14. On the contrast enhanced MRA images, the collateral circulation was sufficient in 3 (5.45%) and 10 (18.18%) cases involving the Sylvian fissure and leptomeningeal convexity collateral vessels respectively. The infarct volume on onset MRI was 43.98 ± 78.13 ml, with a median HV score of 5.09 ± 2.20 points. After endovascular recanalization treatment, successful recanalization to a modified TICI score of ≥ 2b was achieved in 46 (83.64%) cases; The mRS score was 0–2 at 3 months after stroke in 18 (32.73%) cases, but 4 (7.27%) cases had died in 3 months (Table 1).

**Table 1:** Demographic and clinical characteristics, image findings of all patients (n = 55)

Variables	Value
Male sex (%)	33 (60)
Age (years)	68.96 ± 13.54
Hypertension (%)	44 (80)
Diabetes (%)	7 (12.73)
Dyslipidemia (%)	27 (49.09)
Habitual smoker (%)	7 (12.73)
Atrial fibrillation (%)	30 (54.55)
NIHSS score at arrival	12.71 ± 5.14
Onset-to-CT-imaging time (minutes)	102.71 ± 54.01
Onset-to-MRI-imaging time (minutes)	182.31 ± 58.49
Onset-to-DSA-imaging time (minutes)	244.07 ± 70.54
Onset-to-reperfusion time (minutes)	323.91 ± 81.68
Location of symptomatic occlusion (%)	
Middle cerebral artery-M1	25 (45.45)
Middle cerebral artery-M2	7 (12.73)
Intracranial internal cerebral artery	7 (12.73)
Extracranial internal cerebral artery	10 (18.18)
Both middle cerebral artery and internal cerebral artery	6 (10.91)
**Collateral status at the Sylvian fissure (%)**	
Insufficient	52 (94.55)
Sufficient*	3 (5.45)
**Collateral status at the leptomeningeal convexity (%)**	
Insufficient	45 (81.82)
Sufficient*	10 (18.18)
**Recanalization treatment (%)**	
Endovascular recanalization only	20 (36.36)
Combined intravenous and endovascular recanalization	35 (63.64)
Infarct volume on onset MRI (mL)	43.98 ± 78.13
HV	5.09 ± 2.20
Successful recanalization (modified TICI score of ≥ 2b) (%)	46 (83.64)
mRS score of 0–2 at 3 months after stroke (%)	18 (32.73)
Mortality rate at 3 months after stroke (%)	4 (7.27)

Data are mean ± SD, n (%), or median (interquartile range) values.

*A sufficient collateral circulation indicates the collaterals in the ischemic hemisphere ≥ 3 points. NIHSS, National Institutes of Health Stroke Scale, TICI, Thrombolysis in Cerebral Infarction, mRS, modified Rankin Scale. HV, hyperintense vessels.

The baseline clinical characteristics and infarct volume onset of the insufficient collateral circulation at the Sylvian fissure and leptomeningeal convexity according to favorable functional recovery at 3 months after stroke are presented in Table 2. Both of insufficient collateral circulation at the Sylvian fissure and leptomeningeal convexity, the NIHSS score at arrival and preoperative infarct volume on onset MRI were significantly lower in mRS score of 0–2 (both *P* < 0.05). After conventional angiography, successful recanalization (modified TICI score of ≥ 2b) was more frequently observed in the group with mRS score of 0–2, but the differences did not reach statistical significance (at the Sylvian fissure: *P* = 0.102; at the leptomeningeal convexity: *P* = 0.187). Multivariate logistic models showed that age and collateral status at the leptomeningeal convexity were independently affected the functional recovery at 3 months after stroke, while the NIHSS score and infarct volume onset were not significant in predicting the functional recovery after stroke (Table 3).

**Table 2:** Results of bivariate analyses according to functional recovery at 3 months after stroke among subjects with an insufficient collateral circulation

Variable	Insufficient collateral status at the Sylvian fissure (*n* = 52)			Insufficient collateral status at the leptomeningeal convexity (*n* = 45)		
	mRS score of 0–2 (*n* = 16)	mRS score of 3–6 (*n* = 36)	*P*	mRS score of 0–2 (*n* = 11)	mRS score of 3–6 (*n* = 34)	*P*
Male sex (%)	12 (75)	20 (55.56)	0.183	7 (63.64)	19 (55.88)	0.919
Age (years)	64.00 ± 11.92	72.89 ± 10.98	0.011	65.36 ± 11.88	72.12 ± 10.80	0.085
Hypertension (%)	12 (75)	31 (86.11)	0.562	8 (72.73)	30 (88.24)	0.450
Diabetes (%)	3 (18.75)	3 (8.33)	0.539	3 (27.27)	3 (8.82)	0.192
Dyslipidemia (%)	5 (31.25)	20 (55.56)	0.105	3 (27.27)	20 (58.82)	0.069
Habitual smoker (%)	0 (0)	7 (19.44)	0.145	0 (0)	6 (17.65)	0.324
Atrial fibrillation (%)	6 (37.5)	22 (61.11)	0.115	4 (36.36)	20 (58.82)	0.194
NIHSS score at arrival	10.06 ± 4.65	14.25 ± 4.91	0.006	10.36 ± 5.45	14.41 ± 4.99	0.027
Onset-to-CT-imaging time (minutes)	88.88 ± 43.02	105.69 ± 59.05	0.311	91.09 ± 46.68	103.79 ± 58.71	0.518
Onset-to-MRI-imaging time (minutes)	167.69 ± 52.04	187.19 ± 61.53	0.275	180.18 ± 50.36	186.35 ± 62.08	0.767
Onset-to-DSA-imaging time (minutes)	228.56 ± 64.35	247.47 ± 74.60	0.384	236.55 ± 67.31	245.59 ± 76.22	0.727
Onset-to-reperfusion time (minutes)	296.44 ± 82.82	337.00 ± 81.77	0.106	303.27 ± 87.36	338.50 ± 83.51	0.236
**Location of symptomatic occlusion (%)**			0.006			0.020
Middle cerebral artery-M1	7 (43.75)	18 (50)		4 (36.36)	17 (50)	
Middle cerebral artery-M2	0 (0)	6 (16.67)		0 (0)	5 (14.71)	
Intracranial internal cerebral artery	4 (25)	2 (5.56)		2 (18.18)	2 (5.88)	
Extracranial internal cerebral artery	5 (31.25)	4 (11.11)		5 (45.45)	4 (11.76)	
Both middle cerebral artery and internal cerebral artery	0 (0)	6 (16.67)		0 (0)	6 (17.65)	
**Recanalization treatment (%)**			0.429			1
Endovascular recanalization only	6 (37.5)	13 (36.11)		4 (36.36)	12 (35.29)	
Combined intravenous and endovascular recanalization	10 (62.5)	13 (36.11)		7 (63.64)	22 (64.71)	
Infarct volume on onset MRI (mL)	13.61 ± 10.99	59.80 ± 92.74	0.006	11.62 ± 8.58	62.84 ± 94.61	0.004
HV	5.19 ± 2.64	5.14 ± 1.91	0.941	6.00 ± 2.14	5.06 ± 1.94	0.179
Successful recanalization (modified TICI score of ≥ 2b) (%)	16 (100)	28 (77.78)	0.102	11 (100)	26 (76.47)	0.187

HV, Hyperintense vessels, TICI, Thrombolysis in Cerebral Infarction, mRS, modified Rankin Scale.

**Table 3:** Multivariate analysis of the preoperative predictors of functional recovery at 3 months after stroke (n = 55)

Multivariable variable	Odds ratio (95% confidence interval)	*P*
Mode l : age, NIHSS score		
Age	1.079 (1.025–1.136)	0.004
Model 2: model1 + preoperative infarct volume		
Age	1.088 (1.027–1.154)	0.004
Preoperative infarct volume	1.033 (0.988–1.080)	0.154
Model 3: model2 + collateral status		
Age	1.094 (1.025–1.168)	0.007
Collateral status at the leptomeningeal convexity	9.542 (1.812–50.245)	0.008

NIHSS, National Institutes of Health Stroke Scale.

**Figure 1:**
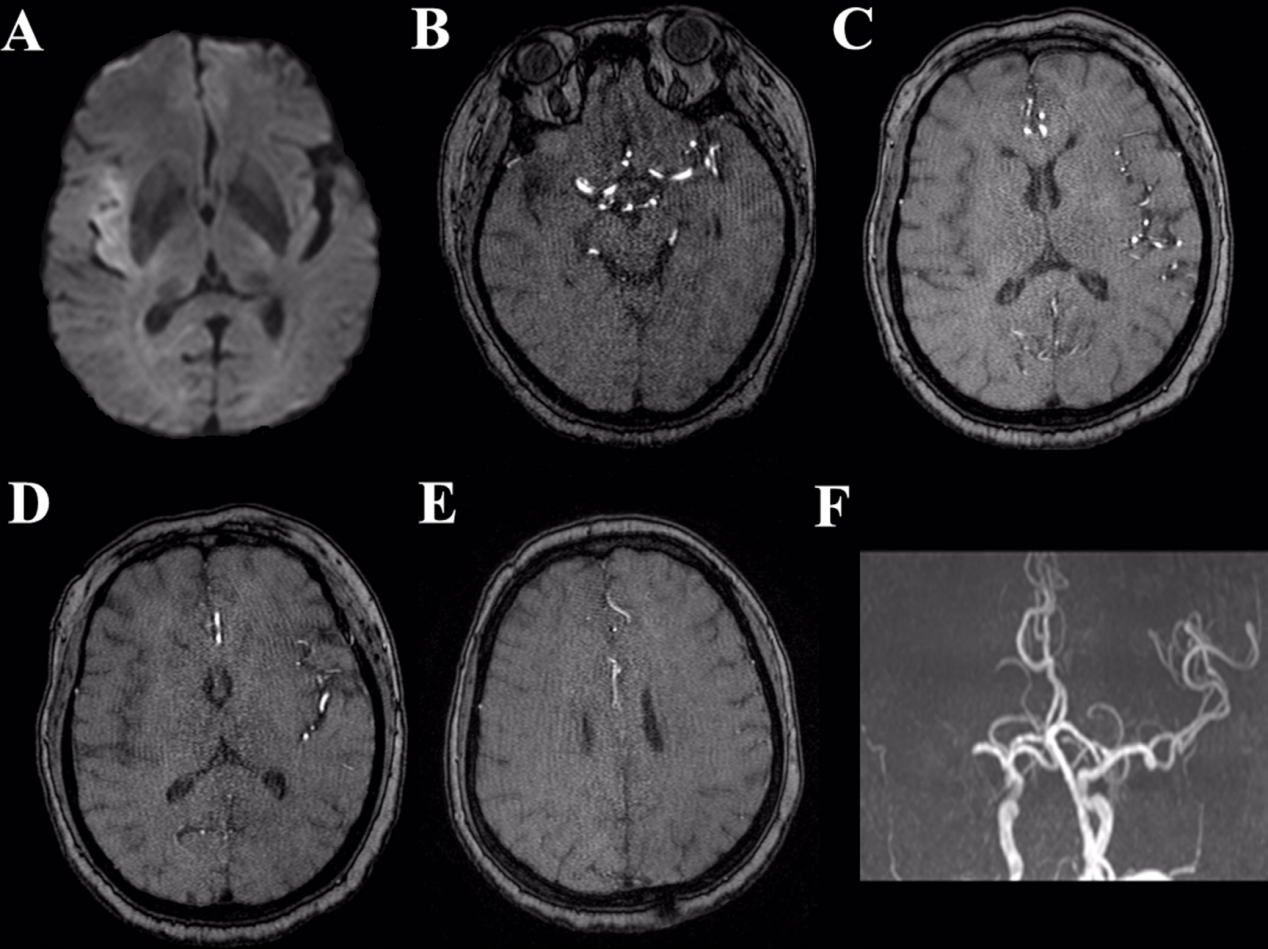
Illustrative case depicting the collateral status from preoperative MRI Preoperative DWI (**A**) shows acute cerebral infarction in the right temporal lobe. Sylvian collateral (**B, C**) poor. Leptomeningeal collateral (**D, E**): poor. 3D-TOF MRA (**F**) shows occlusion of right MCA.

**Figure 2:**
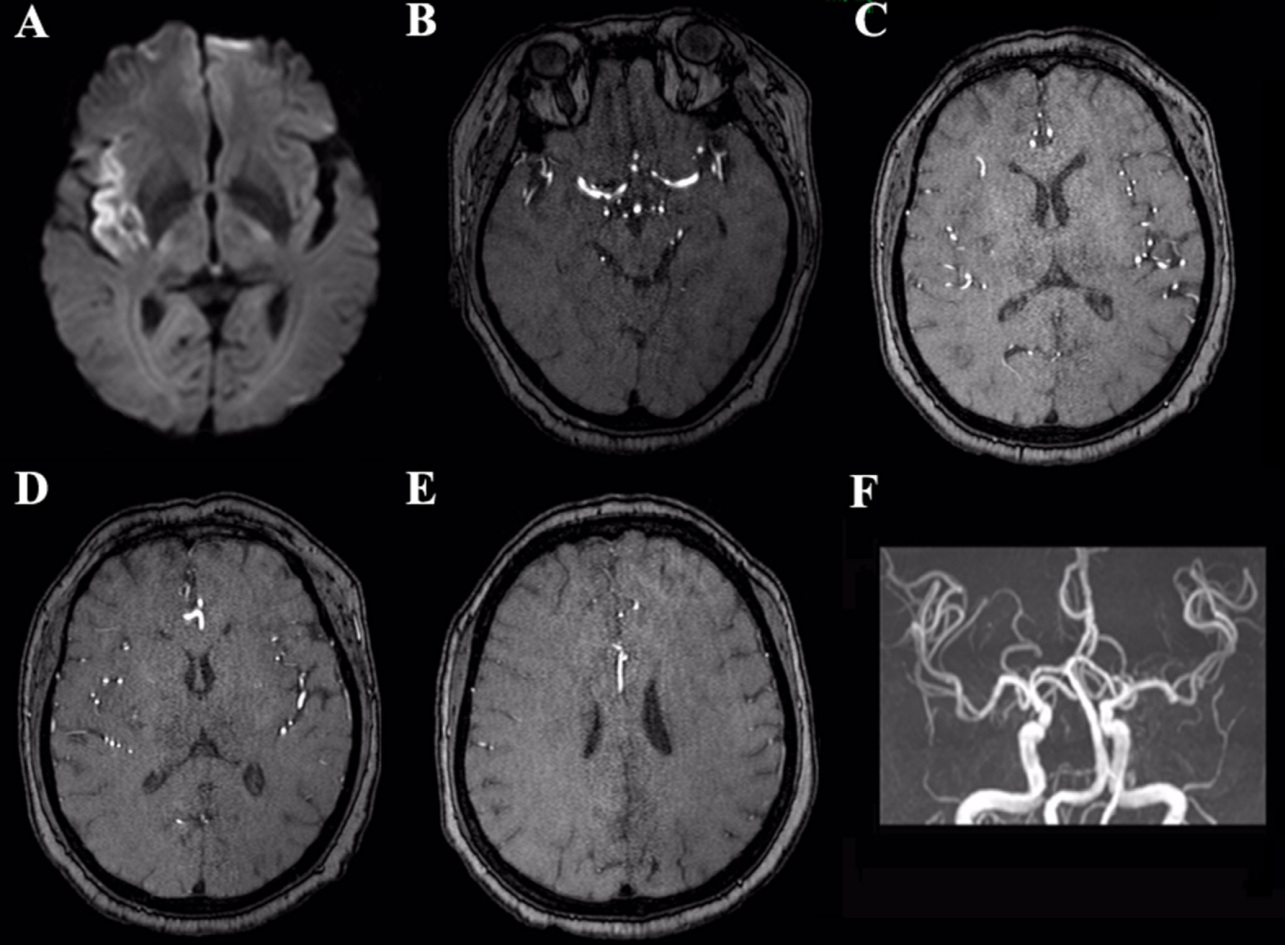
The same case with postoperative MRI imagings (**A–F**). Postoperative DWI (A) demonstrates the lesion mid enlargement. Sylvian collateral (B, C) adequate. Leptomeningeal collateral (D, E) adequate. 3D-TOF MRA (F) shows right MCA normal.

The postoperative infarct volume was slightly larger than that of pre-operation in group with mRS score of 0–2, while in group with mRS score 3–6, the postoperative infarct volume became larger obviously, both groups were statistically different (*P* = 0.039; *P* = 0.002). The HV almost disappeared after operation, especially in the group with mRS score of 0–2 (*P* < 0.001; *P* < 0.001). The postoperative collateral circulation at the Sylvian fissure and leptomeningeal convexity was much better than that in pre-operation (Table 4, Figures 1 and 2). While multivariate logistic models showed that postoperative infarct volume, HV, collateral status at the Sylvian fissure and at the leptomeningeal convexity were not significant in predicting the functional recovery after stroke (Table 5).

**Table 4:** Comparison of facts between pre-operation and post-operation

Variable	mRS score of 0–2 (*n* = 11)			mRS score of 3–6 (*n* = 23)		
	Pre-operation	Post-operation	*P*	Pre-operation	Post-operation	*P*
Infarct volume on MRI (mL)	14.15 ± 10.73	21.39 ± 17.41	0.039	16.88 ± 16.64	57.27 ± 56.67	0.002
HV	5.00 ± 2.90	0.36 ± 0.81	0.000	5.22 ± 1.76	1.74 ± 2.68	0.000
Collateral status at the Sylvian fissure	1.91 ± 0.70	3.00 ± 0.00	0.000	1.83 ± 0.49	2.70 ± 0.47	0.000
Collateral status at the leptomeningeal convexity	2.45 ± 1.21	3.09 ± 0.30	0.089	1.52 ± 0.73	2.48 ± 0.95	0.000

HV: Hyperintense vessels, mRS: modified Rankin Scale.

**Table 5:** Multivariate analysis of the postoperative predictors of functional recovery at 3 months after stroke (n = 34)

Multivariable variable	Odds ratio (95% confidence interval)	*P*
postoperative infarct volume	1.027 (0.990–1.066)	0.154
HV	0.735 (0.279–1.939)	0.534
Collateral status at the Sylvian fissure	0.000	0.999
Collateral status at the leptomeningeal convexity	0.512(0.026–9.996)	0.658

HV, Hyperintense vessels.

## DISCUSSION

From our analysis of 55 AIS patients who were treated with an endovascular recanalization procedure, we found that most of collateral circulation was insufficient, 94.55% and 81.82% of collateral status at the Sylvian fissure and the leptomeningeal convexity respectively. In the patients with insufficient collateral circulation, age, NIHSS score at arrival and infarct volume onset were significant different between mRS score of 0–2 and mRS score of 3–6. The age and collateral status at the leptomeningeal convexity was the predictor for the functional recovery at 3 months after stroke. After operation, the infarct volume became larger, the HV almost disappeared and a majority of the collateral circulation was sufficient.

The collateral circulation seems functionally inactive in the normal and stable brain, after focal cerebral ischemia, cerebral arteries and capillaries in the brain are impaired early [29, 30], collateral blood supply to vessels and brain tissue located within the territory of the occluded artery to minimize the degree of ischemic vascular. Since early collateral development via leptomeningeal anastomoses after MCA occlusion is induced by a pressure gradient between the anterior cerebral artery or PCA territory and a territory distal to the MCA occlusion site [30], these collateral circulation may protect brain tissue and/or vessel structures from ischemic damage until MCA recanalization. Our study suggested that there existed association between collateral circulation and good outcome. Patients with sufficient collateral status at the leptomeningeal convexity showed a trend for better outcomes, odds ratio 9.542 (1.812–50.245). The smaller odds ratio in our study could imply that patients with good collaterals are more favorable, while the collateral status at the Sylvian fissure is not significant association with good outcome. Leptomeningeal collaterals have been considered as a beneficial role in AIS patients [16]. The leptomeningeal collateral circulation can preserve cerebral blood flow in the territory of the occluded artery [30], and well-developed leptomeningeal collateral circulation can maintain the perfusion of penumbral regions and protect distal brain tissues [31, 32]. The prior studies showed that the collateral circulation can predict outcome irrespective of final recanalization status [19, 33, 34], which was consistent with our study that considered sufficient collateral status at the leptomeningeal convexity as a predictor for good clinical outcome.

Another important finding of this study is that the postoperative infarct volume was slightly larger than that of pre-operation in group with mRS score of 0–2, while in group with mRS score 3–6, the postoperative infarct volume became larger obviously. Both groups with mRS score of 0–2 and of 3–6 were statistically different. Son et al. showed that the degree of infarct growth differed depending on the collateral status, regardless of successful recanalization [35]. In our study, the collateral circulation at the Sylvian fissure and leptomeningeal convexity was much better in post-operation, especially in the group with mRS score of 0–2. The results demonstrate that most patients have HV in the insufficient collateral circulation. However, when the collateral circulation is sufficient, the HV almost disappear. These results may be explained by the speed of collateral flow. Distal HV are thought to be linked with stationary blood and slow antegrade collateral or retrograde collateral circulation [36, 37]. Kim et al. thought that if patients with MCA occlusion have sufficiently rapid collaterals from arteries unaffected by occlusion, distal HV beyond the site of arties occlusion may not be identified on FLAIR images [26]. Based on our results, postoperative infarct volume and HV cannot be used to predict the functional recovery.

Several limitations should be noted in the current study. Firstly, this is a retrospective case-control study with a limited sample size that might cause overestimation. More samples were still required to establish the influence of collateral circulation on clinical outcome. Secondly, although the site of occlusion may be associated with the collateral status, we did not analyze it further mainly due to small number of subjects. Moreover, the post-operative infarct volume was measured within 24 hours after operation, it will be better for evaluating the infarct volume change in 7 days and/or one month due to the infarct volume stationary relatively without ischemic penumbra influence.

## CONCLUSIONS

In summary, a sufficient baseline collateral status in acute ischemic stroke patients was associated with improved functional recovery and decreased mortality rate at 3 months after stroke. In addition, the age and collateral status at the leptomeningeal convexity may be useful markers for the prediction of functional recovery.
